# Resolving the Complexity of Ubiquitin Networks

**DOI:** 10.3389/fmolb.2020.00021

**Published:** 2020-02-27

**Authors:** Katarzyna Kliza, Koraljka Husnjak

**Affiliations:** Institute of Biochemistry II, Medical Faculty, Goethe University, Frankfurt, Germany

**Keywords:** ubiquitin, ubiquitin receptor, affinity purification, mass spectrometry, E3 ligase, deubiquitinating enzyme

## Abstract

Ubiquitination regulates nearly all cellular processes by coordinated activity of ubiquitin writers (E1, E2, and E3 enzymes), erasers (deubiquitinating enzymes) and readers (proteins that recognize ubiquitinated proteins by their ubiquitin-binding domains). By differentially modifying cellular proteome and by recognizing these ubiquitin modifications, ubiquitination machinery tightly regulates execution of specific cellular events in space and time. Dynamic and complex ubiquitin architecture, ranging from monoubiquitination, multiple monoubiquitination, eight different modes of homotypic and numerous types of heterogeneous polyubiquitin linkages, enables highly dynamic and complex regulation of cellular processes. We discuss available tools and approaches to study ubiquitin networks, including methods for the identification and quantification of ubiquitin-modified substrates, as well as approaches to quantify the length, abundance, linkage type and architecture of different ubiquitin chains. Furthermore, we also summarize the available approaches for the discovery of novel ubiquitin readers and ubiquitin-binding domains, as well as approaches to monitor and visualize activity of ubiquitin conjugation and deconjugation machineries. We also discuss benefits, drawbacks and limitations of available techniques, as well as what is still needed for detailed spatiotemporal dissection of cellular ubiquitination networks.

## Introduction

Post-translational modifications (PTMs) greatly increase the complexity and functional diversity of the proteome, ensuring rapid and dynamic cellular responses to the environmental and intracellular factors (Walsh et al., 2005). Extensive research over the last few decades has revealed elaborate control of a variety of cellular processes by a small protein ubiquitin (Ub), including cellular proteostasis, DNA repair, trafficking and immunity (Varshavsky, 2006; Kulathu and Komander, 2012).

**FIGURE 1:**
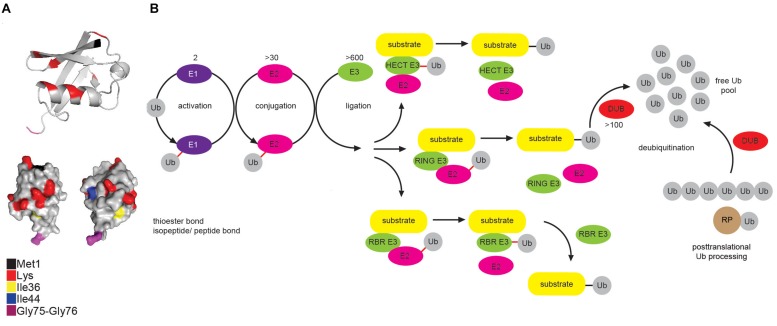
Multicomponent enzymatic machineries assemble and disassemble ubiquitin modification. **(A)** Ub belongs to the β-grasp fold (β-GF) family (Vijay-Kumar et al., 1987), in which β-GF is formed by five-stranded β-sheet, a short 3_10_ helix and a 3.5-turn α-helix. The C-terminal Ub tail is essential for Ub conjugation and hence, for all the Ub functions. Functionally relevant Ub residues are depicted in different colors. The figure was generated from the PDB entry 1UBQ by PyMOL v1.7.6.0 software. **(B)** Coordinated activity of Ub-activating (E1), Ub-conjugating (E2), and Ub-ligating enzyme (E3) is required for Ub attachment to substrate protein. The action modes of the three main groups of E3 ligases (RING, HECT and RBR) are also depicted. In mammals, Ub is encoded by four different genes: UBA52 and RPS27A genes encode a single Ub molecule fused to the ribosomal subunits L40 and S27a, respectively (depicted as RP-Ub), UBB and UBC genes encode 2 different polyUb precursor proteins (exemplified here as Ub_6_ fusion). More than 100 cellular DUBs process newly translated Ub-containing polypeptides, remove Ub from modified substrates and disassemble unanchored Ub chains.

Ub is a small, highly compact globular protein, with the exception of its unrestrained and flexible C-terminal tail (Figure 1A). To achieve high cellular Ub concentrations, 4 different genes (UBB, UBC, RPS27, and UBA52) encode Ub in mammals. Genes UBB and UBC encode linear fusions of 3 and 9 Ub molecules, respectively, whereas RPS27A and UBA52 encode Ub as in-frame fusion to a small and large ribosomal protein, respectively (Figure 1B) (Ozkaynak et al., 1984; Finley et al., 1989).

Protein modification by Ub (ubiquitination) occurs through the formation of the covalent bond between α–carboxyl group of the terminal glycine (Gly) residue of Ub and, typically, ε-amino group of an internal lysine (Lys) residue of the substrate. Interestingly, some mammalian and viral E3 ligases target thiol group of cysteine (Cys) residue (Cadwell and Coscoy, 2005; Williams et al., 2007), whereas a subset of substrates, such as ataxin-3 and tau, is modified by the attachment of Ub to an α-amino group of their N-terminal residues, in a process known as N-terminal ubiquitination (Ciechanover and Ben-Saadon, 2004). Additionally, serine (Ser) and threonine (Thr) residues can also function as ubiquitination sites, forming hydroxyester bonds between Ub and target proteins (McDowell and Philpott, 2016) and thus expanding the biological importance of ubiquitination even further.

Ubiquitination is achieved by a coordinated and sequential enzymatic cascade (Figure 1B). Classically, Ub is activated in an ATP-dependent reaction by an Ub-activating (E1) enzyme and subsequently transferred to the active Cys residue of an Ub-conjugating (E2) enzyme, followed by Ub attachment to a substrate mediated by an Ub-ligating (E3) enzyme. Until now, two E1s, nearly 30 E2s and over 600 E3s have been identified in humans. Mechanistically, E3 ligases belong to either RING (really interesting new gene), HECT (homologous to E6-AP C terminus or RBR (RING-between-RING, hybrid RING-HECT) classes and can generate Ub linkages of different length and architecture (Metzger et al., 2012; Walden and Rittinger, 2018).

The activity of ubiquitination machinery can be reversed by more than 100 deubiquitinating enzymes (DUBs), which hydrolyze isopeptide or peptide bond resulting in Ub deconjugation from the ubiquitinated protein (Figure 1B) (Komander et al., 2009; Mevissen and Komander, 2017). DUBs affect cellular pool of free Ub by releasing newly synthesized Ub from Ub precursors, removing non-essential Ub molecules and recycling Ub from the former ubiquitination events (Reyes-Turcu et al., 2009; Grou et al., 2015).

### Different Forms of Ubiquitin Modifications Exist in Nature

**FIGURE 2:**
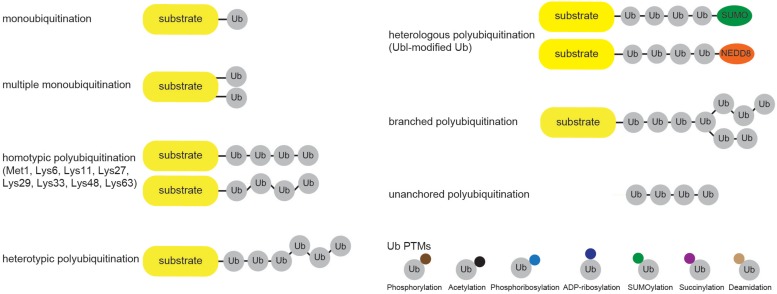
Cellular ubiquitin modification comes in different formats. Single Ub moieties can modify proteins at one (monoubiquitination) or several (multiple monoubiquitination) Lys residues. Ub can form eight distinctive homotypic linkages, either through Met1 (linear Ub chain) or 7 internal Lys residues (Lys6-, Lys11-, Lys27-, Lys29-, Lys33-, Lys48-, and Lys63-linked Ub chains). Additional complexity is achieved through the formation of heterotypic Ub chains, which contain multiple Ub linkages and adopt mixed or branched topology. Furthermore, heterologous polymers contain additional UBLs, such as SUMO or NEDD8, within Ub chains. Ub molecules undergo various PTMs, including phosphorylation and acetylation, which regulate their binding properties and abilities to generate Ub chains.

Cellular Ub modifications occur in various forms, which are usually referred to as “Ub code.” Modification by a single Ub moiety (monoubiquitination) is the most abundant Ub modification that regulates DNA repair, transcription, signal transduction, viral budding, endocytosis and even proteasomal degradation (Chen and Mallampalli, 2009; Braten et al., 2016). After Ub is transferred to the ε-amino group of a target Lys, any of the eight amino groups of Ub (Met1, Lys6, Lys11, Lys27, Lys29, Lys33, Lys48, Lys63) can be attached to the C terminus of another Ub to form Ub chain of variable length, linkage type and configuration (homo- and heterotypic/branched Ub chains). Even though functional significance of several Ub modifications (such as Lys48- and Lys63-linked ubiquitination) is largely known, the biological significance of other Ub modifications is still far from being fully understood (Figure 2). Amongst the homotypic Ub chains, Lys48-linked Ub polymers were historically first identified and are predominant among homotypic polyUb chains (Peng et al., 2003; Swatek and Komander, 2016). These Ub linkages mark proteins for proteolytic degradation, which in turn regulates signal transduction, cell division, stress response, adaptive immune system and development (Hershko and Ciechanover, 1998; Wang and Maldonado, 2006; Park et al., 2007). The remaining homotypic polyUb chains are collectively called atypical (Liu et al., 2015; Swatek and Komander, 2016), and their physiological roles are nicely summarized elsewhere (Kulathu and Komander, 2012; Akutsu et al., 2016). Branched Lys11/Lys48 and hybrid Met1/Lys63 linkages were recently implicated in proteasomal degradation and NFκB signaling, respectively (Emmerich et al., 2013; Meyer and Rape, 2014; Grice et al., 2015).

Besides by ubiquitination, Ub molecules can also be modified by acetylation, phosphorylation, ADP-ribosylation, phosphoribosylation, deamidation, SUMOylation and succinylation (Figure 2). Ub acetylation negatively regulates Ub chain elongation by competing with ubiquitination to regulate the stability of target proteins (Ohtake et al., 2015). By using mass spectrometry (MS) approach, Ohtake et al. (2015) identified acetylation of endogenous Ub at residues Lys6, Lys48, and Lys63. Since the same Ub residues are involved in Ub chain formation, it is not surprising that acetylation inhibits Lys11-, Lys48-, and Lys63-linked polyUb chain elongation by several E2 enzymes *in vitro*, without significantly affecting E1 and E2 charging or substrate monoubiquitination (Ohtake et al., 2015). Moreover, two of the major histones, H2A and H2B, were proposed as substrates for acetylated monoUb. Ser/Thr kinase PINK1 accumulates on depolarized mitochondria upon decrease in mitochondrial membrane potential and phosphorylates N-terminal Ub-like (UBL) domain of E3 ligase PARKIN (Kondapalli et al., 2012; Shiba-Fukushima et al., 2012; Kazlauskaite et al., 2014; Okatsu et al., 2018), as well as Ub itself (Kane et al., 2014; Koyano et al., 2014). Both modifications occur at the homologous position (Ser65) in PARKIN UBL domain and Ub. PARKIN UBL domain keeps PARKIN in autoinhibited state (Chaugule et al., 2011), and phosphorylated Ub is sufficient to allosterically activate it by unlocking its autoinhibition (Kane et al., 2014; Koyano et al., 2014; Wauer et al., 2015a). This topic was recently reviewed in great detail in Herhaus and Dikic (2015) and Swatek and Komander (2016). Interestingly, phosphorylation at Ser65 affects Ub structure, E2 discharging and formation of Ub chains by a subset of E2 and E3 enzymes, such as CDC34, UBC13/UEV1A, TRAF6 and HOIP (Wauer et al., 2015b). Some DUBs are also impaired in hydrolyzing Ser65-phosphoUb-containing chains (Wauer et al., 2015b). Even though other residues in Ub molecule have also been reported to be phosphorylated in various MS screens, the physiological significance of these modifications is not yet known.

Yang et al. (2017) have recently shown that Ub can undergo NAD^+^-, E1- and E2-dependent monoADP-ribosylation. The process is catalyzed by a heterodimer of ADP-ribosyltransferase PARP9 and histone E3 ligase DTX3L. Since ADP-ribose is attached to the C-terminal Gly residue of Ub, monoADP-ribosylation of Ub prevents Ub conjugation and consequently impedes the Ub ligase activity and the function of DTX3L in non-homologous end joining (NHEJ) DNA repair pathway (Yang et al., 2017).

Many pathogens have evolved intricate mechanisms to hijack Ub system of the host, often mimicking components of the host Ub system, such as E3 ligases (Maculins et al., 2016) and DUBs (Pruneda et al., 2016). Recently discovered *Legionella pneumophila* effector SdeA utilizes unique, ATP-independent and NAD^+^-dependent ubiquitination mechanism that does not involve host E1 and E2 enzymes (Qiu et al., 2016). SdeA possesses intrinsic monoADP-ribosyltransferase and phosphodiesterase activities, which enable intermediate ADP-ribosylation and subsequent phosphoribosylation of Ub Arg40 residue. SdeA subsequently mediates ubiquitination of the target protein by conjugating phosphoribosylated Ub to Ser residue of the substrate through phosphodiester bond (Bhogaraju et al., 2016). Several proteins were shown to be ubiquitinated by SdeA, including small GTPase Rab33b and ER component RTN4 (Qiu et al., 2016; Kotewicz et al., 2017). Ser-linked ubiquitination of those proteins affects their cellular functions. Since SdeA-mediated ADP-ribosylation and phosphoribosylation of Ub inhibit activation of E1 and E2 enzymes, they also impair a plethora of essential Ub-dependent cellular processes, such as proteasomal degradation and mitophagy (Bhogaraju et al., 2016).

Cif proteins from enteropathogenic *Escherichia coli* (*E. coli*) (EPEC) and *Burkholderia pseudomallei* bacteria belong to the group of bacterial effectors targeting Ub signaling by catalyzing deamidatation of Ub at residue Gln40, which inhibits polyUb chain formation (Cui et al., 2010).

Recent proteomic studies have also revealed Ub modification by a small Ub-like modifier (SUMO) at multiple Lys residues (Galisson et al., 2011; Lamoliatte et al., 2013; Hendriks et al., 2014). Additionally, ubiquitinated SUMO has also been reported (Lamoliatte et al., 2013; Hendriks et al., 2014).

### Ubiquitin Readers Decode Ubiquitin Code and Induce Specific Cellular Responses

Ub code is recognized by proteins containing single or multiple Ub-binding domains (UBDs), referred to as Ub readers or decoders that, more or less specifically, recognize Ub chain topology and length and enable execution of specific cellular processes (Husnjak and Dikic, 2012).

**FIGURE 3:**
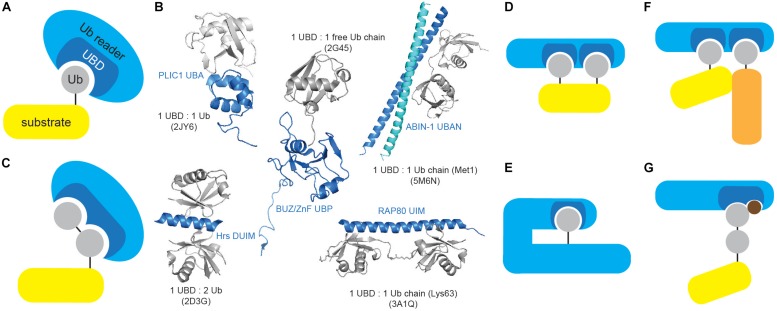
Ubiquitin-binding domains come in different shapes and forms. **(A)** Ub receptors contain single or multiple (identical or different) motifs or domains that non-covalently bind Ub or Ub chains. **(B)** UBDs differ in shape and Ub/Ub chain specificity. Several UBDs in complex with Ub or Ub chains are depicted: PLIC1 UBA (Ub-associated, PDB code: 2JY6), Hrs DUIM (double-sided Ub-interacting motif, PDB code: 2D3G), ZnF UBP/BUZ (zinc-finger Ub-binding, PDB code: 2G45), ABIN-1 UBAN (Ub-binding domain in ABINs and NEMO, PDB code: 5M6N) and RAP80 tandem UIMs (Ub-interacting motif, PDB code: 3A1Q). Ub and UBD structures are depicted in gray and blue, respectively. The figure was generated from PDB entries by PyMOL v1.7.6.0 software. **(C)** A subset of UBDs recognizes specific types of Ub modifications, such as specific Ub linkages. **(D)** Cooperative recognition of Ub modifications by two or more UBDs is one of many approaches to increase avidity of Ub:UBD interaction. **(E)** Monoubiquitinated proteins can bind their intrinsic UBDs to regulate their function. The interaction between Ub modification and UBD (on the same protein) provides an efficient switch between active and inactive Ub receptor conformation. **(F)** Ub:UBD interactions often lead to formation of large protein complexes. Most of the Ub:UBD interactions are relatively weak. Multiple UBDs, due to avidity, contribute to the strengthened interaction between Ub and UBDs. Such multiple UBDs and Ub modifications enable formation of highly dynamic protein complexes. **(G)** A subset of UBDs could potentially recognize specific PTM-modified Ub modifications.

Ub readers interact with their targets in a transient, non-covalent way (Figure 3A) and are often found in a complex with E3 ligases and DUBs, where UBDs contribute to enzyme functionality and/or substrate selectivity, exemplified by the functional coupling between proteasomal Ub receptor RPN13 and DUB UCH37 (Reyes-Turcu and Wilkinson, 2009). Moreover, intrinsic UBDs often determine functionality of ubiquitinating and deubiquitinating enzymes.

UBDs utilize diverse surfaces to contact Ub or Ub polymers, which usually engage confined areas to interact with UBDs. Ub:UBD binding induces mild conformational changes in Ub surface, providing optimal Ub:UBD interface. Although majority of Ub surface is polar, it possesses few hydrophobic patches essential for Ub:UBD interaction, including the most frequently utilized Ile44/Val70 patch and the less common Ile36 and Phe4 patches (Sloper-Mould et al., 2001; Winget and Mayor, 2010). Another non-canonical hydrophobic area centered on Leu8 was identified in members of Y-family translesion synthesis (TLS) polymerases (Bienko et al., 2005). Interestingly, C-terminal part of Ub serves as a binding surface for DUB USP5/IsoT and assists in cleaving unanchored polyUb chains (Reyes-Turcu et al., 2006).

UBDs are typically independently folded, modular domains of up to 150 amino acids and with remarkable structural heterogeneity that can accommodate a large number of known Ub modifications. UBDs have been classified into nearly 25 subfamilies based on adapted structural folds, which can be divided into helical (i.e., Ub-associated, UBA; Ub-interacting motif, UIM), zinc finger (ZnF), Ub-conjugating-like, pleckstrin homology (PH) and other domains (Table 1 and Figure 3B). Interestingly, not all the members of a specific UBD family can bind Ub, as exemplified by SH3 (Stamenova et al., 2007) and CUE (Lim et al., 2019) domains.

**TABLE 1:** List of currently known ubiquitin-binding domains.

**Domains**	**Abbreviation**	**Name**	**Examples (proteins with specific UBD)**
Helical	UIM	Ub-interacting motif	RPN10, VPS27, USP28, ATAXIN-3, EPS15, STAM1, STAM2, RAP80, DNAJB2, USP37, USP25, EPSINs
	MIU	Motif interacting with Ub	Rabex-5, RNF168
	UMI	UIM- and MIU-related UBD	RNF168
	DUIM	Double-sided UIM	HRS
	UBA	Ub-associated domain	PLIC1/2, HHR23A/B, p62, NBR1, Cbl-b, USP5, UBC1, HERC2, Vps13D, USP25
	CUE	Coupling of Ub to ER degradation domain	Cue2, Vps9
	GAT	GGA and TOM domain	GGA1, GGA2, GGA3, TOM1
	UBAN	Ub-binding domain in ABINs and NEMO	ABIN1, ABIN2, ABIN3, NEMO, OPTN
	VHS	VPS27, HRS and STA domain	VPS27, HRS, STAM1, STAM2, GGA1, GGA2, GGA3, TOM1
	UBM	Ub-binding motif	Polymerase iota, Rev1
	MyUb	Myosin VI UBD	Myosin VI
	AnkUBD	Ankyrin (Ank) repeat UBD	TRABID
Zinc finger (ZnF)	UBZ	Ub-binding ZnF domain	TAX1BP1, Polymerase eta, WRNIP1, FAAP20
	NZF	Npl4 ZnF domain	Npl4, Vps36, TAB2, TAB3, HOIP, HOIL-1L, SHARPIN
	ZnF A20	ZnF of A20 domain	A20, Rabex-5
	ZnF UBP (PAZ, BUZ)	ZnF of Ub-specific processing protease domain	USP5, USP20, HDAC6, BRAP2
Ub-conjugating-like	UBC	Ub-conjugating domain	UbcH5c
	UEV	Ub E2 variant domain	TSG101, Mms2
Pleckstrin-homology (PH)	GLUE	GRAM-like Ub-binding in EAP45 domain	Eap45
	PRU	Pleckstrin-like receptor for Ub	Rpn13
Others		Jab1/MPN domain	Prp8
	PFU	PLAA family UBD	Doa1, PLAA
		SH3, variant	Sla1, CIN85, amphiphysin
		WD40 repeat β-propeller	Doa1, PLAA, Fbxw8, Met30, WDR61, PAF, WDR5
		DC-UbP_N	UBTD2
		MDA-9 UBD	MDA-9

Moreover, a subset of Ub readers with no obvious, structurally defined UBDs has been identified, including intrinsically disordered protein Dss1/SEM1 that binds Ub by binding sites characterized by acidic and hydrophobic residues (Paraskevopoulos et al., 2014). As such “Ub-binding activities” are hard to predict both structurally and bioinformatically, it is unclear how many of such proteins are yet to be identified.

The low-affinity interactions between UBDs and Ub are critical for rapid, timely and reversible cellular responses to a particular stimulus. However, specificity and amplification of Ub binding are required for effective and timely transmission of biological information. This is achieved by a number of different strategies. Approaches toward preferential recognition of various Ub linkages include: existence of Ub linkage-selective UBDs (Figure 3C), differential Ub recognition by UBDs with multiple Ub-binding surfaces, Ub chain specificity induced by UBD dimerization or through UBD conformational adaptation, as well as through contribution of sequences situated outside UBDs to Ub binding. On the other hand, increased avidity of Ub:UBD interaction is achieved by implementing various strategies, such as: ability of UBDs to sense Ub chain length, cooperative Ub binding by tandem of identical or combination of different UBDs (Figure 3D), regulation of accessibility of Ub-binding modules (through inter- and intramolecular interactions and steric hindrance) (Figure 3E), multimerization of Ub-modified proteins and/or Ub receptors (Figure 3F) and coupled ubiquitination of UBD-containing proteins (Husnjak and Dikic, 2012; Rahighi and Dikic, 2012).

### Post-translational Modifications of Ubiquitin Receptors Affect Their Interactions With Ubiquitin

Ub receptors undergo PTMs that modify their affinity to Ub (Figure 3G). Phosphorylation of selective autophagy receptor p62/SQSTM1 UBA domain (at Ser403) by casein kinase 2 (CK2) and innate immunity regulator tank-binding kinase 1 (TBK1) increases its affinity toward Ub and regulates autophagic clearance of ubiquitinated proteins and pathogens (Matsumoto et al., 2011; Pilli et al., 2012). Furthermore, TBK1 also phosphorylates other autophagy receptors, including OPTINEURIN (OPTN), NDP52 (CALCOCO2) and TAX1BP1 (Richter et al., 2016). Phosphorylation of OPTN UBD (Ub-binding domain in ABIN proteins and NEMO; UBAN) at residue Ser473 increases its binding capacity to various Ub chains and enables binding to Ser65-pUb chains, implicating OPTN in PINK1-driven PARKIN-independent mitophagy (Richter et al., 2016).

## Sensitive Tools Help Dissect Cellular Processes Regulated by Ubiquitin System

**FIGURE 4:**
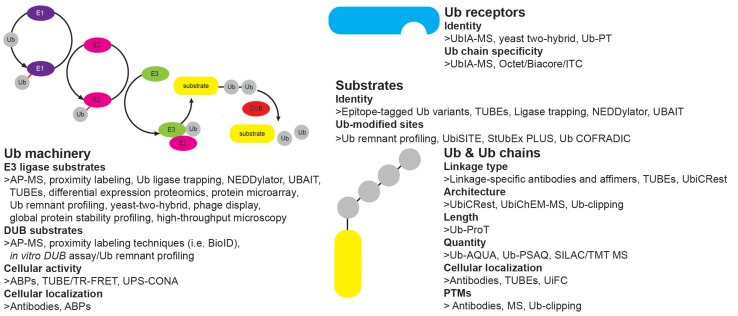
Tools to study ubiquitin system. Overview of different approaches to study features of Ub signaling: E3 ligase and DUB enzymes (enzyme abundance, activity, cellular localization), Ub chains (type, architecture, length, quantity, cellular localization, PTMs), ubiquitinated substrates (identity, modification site, type of modification) and Ub receptors (identity, Ub linkage preference).

In order to study spatiotemporal organization and dynamics of the Ub system, a set of powerful tools has been developed in the last decade, ranging from approaches that study Ub covalent targets, as well as non-covalent “executors” of Ub modifications. Moreover, recent advancement in techniques that enable measurement of the enzymatic activities within Ub system has significantly improved our understanding of the physiological significance of the Ub system (Figure 4).

### Methods to Study Covalent Modifications by Ubiquitin

#### Identification of Substrates Modified by Ubiquitin and Ubiquitin Chains

**FIGURE 5:**
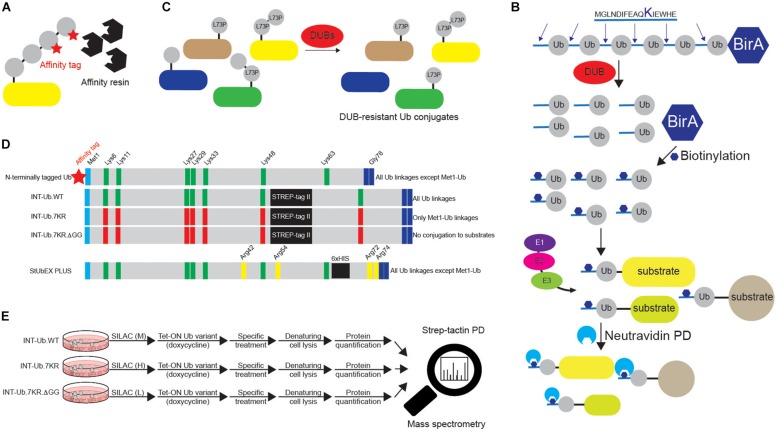
Various approaches to study ubiquitination targets. **(A)** Various N-terminally tagged Ub variants can be exogenously added to cells to enable affinity purifications of ubiquitinated substrates upon denaturing lysis. Short and relatively linear tags (such as 6xHIS, STREP, HA and FLAG), combined with their respective affinity resins (Ni-NTA, strep-tactin, HA agarose and FLAG agarose) are often used in such experiments. Besides N-terminally tagged wild-type Ub, additional Ub variants are often used, such as specific Lys mutants (single or multiple Lys6, Lys11, Lys27, Lys29, Lys33, Lys48 and Lys63 residues mutated to either Arg or Ala). Additionally, residues relevant for Ub binding properties, such as Ile44 and Ile36, can also be mutated to either Arg or Ala. **(B)** Dual BirA system contains synthetic hexaUb sequence fused C-terminally to *E. coli* BirA gene (Ub_6_-BirA). Each Ub in the construct contains 16 amino acids sequence at the N terminus that can be biotinylated by BirA. Once expressed in cells (or organisms), linear hexaUb is processed by cellular DUBs and undergoes biotinylation by BirA. When used by cellular ubiquitination machinery, biotin-containing Ub conjugates can be efficiently affinity purified with neutravidin resins and subsequently analyzed by Western blot and mass spectrometry. Due to the N-terminal tagging of Ub, such approach cannot be used to enrich linear ubiquitination targets. **(C)** DUB-resistant Ub variant Leu73Pro increases the half-life and stability of the formed Ub linkages and facilitates their subsequent identification. **(D)** Two MS-coupled approaches rely on the use of internally tagged Ub variants. Unlike N-terminally tagged Ub, INT-Ub and INT-Ub.7KR variants enable affinity purification of ubiquitinated proteins and Met1-Ub-modified substrates, respectively, due to the existence of internal affinity purification STREP-tag between Ub residues Lys48 and Lys63 that keeps Ub Met1 free to interact with Gly76 of another Ub molecule. **(E)** INT-Ub approach is based on inducible expression of INT-Ub variants in SILAC-treated cells, followed by denaturing lysis, strep-tactin pull-down and subsequent MS analysis. The presence of the internal tag does not affect the overall behavior of Ub. Similar to that, 6xHIS insertion near C-terminus of Ub in StUbEx PLUS approach enables enrichment of HIS-Ub-modified substrates under denaturing conditions. The latter approach can be combined with Ub remnant profiling to identify Ub-modified substrate sites.

Detection and characterization of Ub targets are often challenging due to typically small fraction of a specific protein being modified by Ub, as well as due to highly dynamic nature of Ub modifications. Several techniques enable enrichment and identification of ubiquitinated proteins. Among those, the most common method utilizes transient or ectopic expression of N-terminal epitope-tagged Ub variants, which can be directly conjugated to the substrates as monomers or incorporated into Lys-linked Ub polymers. The cellular epitope-tagged Ub conjugates are then enriched by affinity purification (AP) (Figure 5A). The original proteomic study identified 110 ubiquitination sites in 72 Ub targets isolated from Ub-deficient strain of *S. cerevisiae* expressing 6xHIS-Ub (Peng et al., 2003). Similar strategy enabled detection of 669 Ub-modified human proteins and 44 ubiquitinated peptides in HeLa cell line (Meierhofer et al., 2008). The use of 6xHIS tag enables protein purification under denaturing conditions, thus promoting disassembly of protein complexes and inhibition of DUB activity. Due to the existence of polyHIS stretches within eukaryotic proteins, alternative tags, such as STREP, have also been developed (Danielsen et al., 2011). Another technology takes advantage of the strong biotin:avidin and biotin:neutravidin interactions and is based on the existence of biotinylatable motifs (Figure 5B) (Franco et al., 2011; Lectez et al., 2014). Here, an N-terminal, 16-amino acids biotin-accepting tag is fused to Ub in tandem with *E. coli* biotin ligase BirA. Upon biotin treatment of cells, such Ub variant can be recognized and biotinylated by BirA, followed by AP. The strategy allows *in vitro* and *in vivo* identification of high and low abundant proteins and minimization of false positive hits due to very stringent denaturing conditions (Franco et al., 2011). Proteomic analysis of *in vivo* biotinylated Ub enabled detection of 48 neuronal Ub conjugates from *Drosophila melanogaster* (*D. melanogaster*) embryos, as well as 393 specific ubiquitinated substrates from mouse liver (Franco et al., 2011; Lectez et al., 2014).

The observation that mutation of Ub residue Leu73 to Pro renders polyUb chains resistant to proteolytic cleavage by numerous DUB families led to generation of epitope-tagged Ub Leu73Pro variant that enables purification of stabilized Ub conjugates from cellular extracts and their subsequent proteomics-based identification (Figure 5C) (Bekes et al., 2013). Moreover, Ub and its single Lys variants, in which specific Lys residues are mutated to non-ubiquitinatable amino acids (either Arg or Ala), are frequently used to confirm ubiquitination of protein of interest and to determine the type of conjugated Ub linkage(s) (Kirisako et al., 2006; Kim and Huibregtse, 2009). Finally, since N-terminal tagging abolishes the ability of Ub to form Met1-linked (linear) Ub chains, recently developed Lys-less, internally STREP II-tagged Ub (INT-Ub.7KR) has been successfully used for the MS-based AP (AP-MS) of many novel linear Ub targets (Figures 5D,E) (Kliza et al., 2017).

The abovementioned methods can be combined with either MS or traditional Western blotting.

#### Identification of Substrates and Their Ubiquitin-Modified Sites

**FIGURE 6:**
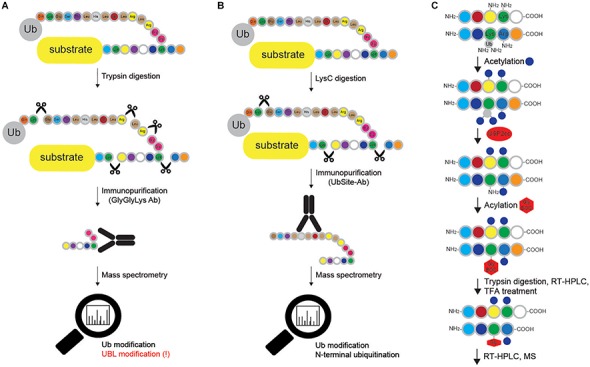
Methods for mapping ubiquitination sites in proteins. **(A)** Ub remnant profiling is based on trypsin digestion of the proteome (cells are previously lysed in urea-containing buffer) combined with immunoprecipitation with monoclonal antibody raised against Lys-ε-Gly-Gly motif that remains on ubiquitinated substrate after trypsin cleavage. Samples are further processed and analyzed by LC-MS/MS. Such approach does not distinguish between modifications by Ub and other UBLs (such as NEDD8 and ISG15), and cannot be applied for Met1- and N-terminally Ub-modified proteins. **(B)** UbiSite antibody recognizes the last 13 amino acids of Ub that remain attached to ubiquitinated proteins upon LysC cleavage. Enriched ubiquitinated proteins are further analyzed by MS. Even though UbiSite approach distinguishes between modifications by Ub and other UBLs, it cannot be used for studying linear ubiquitination, as it does not recognize the signature peptide of linear ubiquitination after tryptic cleavage: Gly-Gly-Met-Gln-Ile-Phe-Val-Lys. **(C)** Ub-COFRADIC approach distinguishes between free (α or ε) and modified primary amines to enable identification of ubiquitinated Lys residues. Initial acetylation by NHS-acetate is only possible on free amines, leaving ubiquitinated Lys residues non-acetylated. Subsequent addition of USP2cc removes all the Ub moieties from Lys residues and enables the attachment of Gly linked to a hydrophobic tert-butyloxycarbonyl (Gly-BOC tag) to previously non-acetylated Lys residues. Trypsin then cleaves C-terminally of Arg residues (but not C-terminally of acetylated Lys). Peptides collected after the first reversed phase (RP)-HPLC run are treated with TFA to remove BOC groups, followed by additional RP-HPLC and MS. In enzyme setting during MS data analysis, ArgC (and not trypsin) should be selected, as cleavage after Lys residues is blocked.

Since MS enables simultaneous identification of Ub-modified proteins and precise mapping of ubiquitination sites on these proteins, several approaches were specifically designed for MS-based identification of ubiquitinated proteins. Serine protease trypsin cleaves Ub after residue Arg74, leaving a diGly remnant from the C terminus of Ub covalently attached to the ubiquitinated Lys residue (“Ub remnant peptide”), thus allowing localization of Ub modification (Xu et al., 2010). Ub remnant profiling (Figure 6A) is a widely used immunopurification method for the identification of Lys ubiquitination sites by MS that exploits monoclonal antibody for selective enrichment of tryptic peptides containing Lys residue with diGly adduct (Xu et al., 2010; Kim et al., 2011; Wagner et al., 2011). Despite its substantial input in proteomic analysis of ubiquitinome, Ub remnant profiling has several limitations, such as additional enrichment of diGly-remnant peptides derived by tryptic digestion of UBL modifiers ISG15 and NEDD8, bias toward amino acid sequence of remnant peptides and inability to recognize linear Ub signature peptide.

Blagoy Blagoev’s group has developed the StUbEx PLUS technique, which overcomes two drawbacks of Ub remnant profiling: recognition of UBL proteins and remnant peptide amino acid sequence preference (Akimov et al., 2018b). To detect ubiquitination sites, the method utilizes internally 6xHIS-tagged Ub in the endogenous Ub knockdown background (Akimov et al., 2018b). Insertion of 6xHIS tag near the C terminus of Ub enables enrichment of HIS-Ub-modified substrates (Figure 5D). Subsequent proteolytic cleavage after Lys residues generates ubiquitinated peptides, which can be detected by MS. In a proof of concept experiment, StUbEx PLUS identified over 41,000 unique diGly-Ub remnant peptides in nearly 7,800 Ub targets in U2OS cells upon proteasome inhibition (Akimov et al., 2018b). However, StUbEx PLUS is more laborious technique than Ub remnant profiling.

The same group has recently developed UbiSite antibody (Figure 6B), which shows significantly improved specificity toward ubiquitinated peptides, since it recognizes the C-terminal 13 amino acids of Ub that remain attached to modified peptides after proteolytic digestion with the endoproteinase LysC (Akimov et al., 2018a). Importantly, the antibody also allows detection of N-terminal ubiquitination and has enabled identification of over 63,000 unique ubiquitination sites on 9,200 proteins in two human cell lines (Akimov et al., 2018a). Nevertheless, just like Ub remnant profiling and StUbEx PLUS, it cannot recognize linear Ub signature peptide.

Noteworthy, in all of the MS-based approaches, sample preparation is critical for reliable identification of residues covalently modified by Ub. The iodoacetamide (IAA) is an alkylating chemical compound commonly used to block Cys residues in sample digestion procedures. However, IAA is not suitable for identification of ubiquitinated protein residues, as it can additionally react with unmodified Lys residues, leaving a modification of the same mass as a diGly remnant, thus mimicking ubiquitination site (Nielsen et al., 2008). Another alkylating agent, chloroacetamide, is therefore recommended for proteomic discovery of ubiquitination sites.

Combined fractional diagonal chromatography (Ub-COFRADIC) is a sensitive alternative approach for identification of ubiquitination sites, initially described in *Arabidopsis thaliana (A. thaliana)* cells (Figure 6C). This multi-step method exploits chemical modification of free primary amines by acetyl groups, which blocks unmodified Lys residues and leaves ubiquitinated Lys residues unmodified. The subsequent deubiquitination by catalytic core of USP2 (USP2cc) exposes the now free amine groups on previously ubiquitinated Lys residues and enables the attachment of Gly-BOC tags to non-acetylated Lys residues. Trypsin digestion of such modified proteins leads to proteolytic cleavage at the C-terminus of Arg, but not Lys residues. After reverse phase HPLC, fractions containing peptides are collected and treated with trifluoroacetic acid (TFA) to remove the BOC groups. Consequently, the residues previously targeted by Ub are now marked by the presence of Gly residues, which can be identified by MS. This proteomics-based approach enabled identification of 3,009 ubiquitination sites on 1,607 plant proteins (Walton et al., 2016). Alike Ub remnant profiling, UbiSite and StUbEx PLUS, Ub COFRADIC is limited to MS studies and is incompatible with standard validation techniques, such as Western blotting and immunofluorescence. Noteworthy, this method generates relatively large peptides, which make MS identification more challenging.

#### Assessment of Ubiquitin Linkage Type, Chain Size and Architecture

Antibodies specifically recognizing Ub modifications are yet another type of reagents for identification of ubiquitinated proteins. While some antibodies detect all Ub-modified proteins (FK2 antibody) (Fujimuro et al., 1994), the others were engineered to selectively recognize single or a subset of specific Ub modifications, such as polyUb chain-specific FK1 antibody (Fujimuro et al., 1994). Moreover, Ub chain topology can be determined by several Ub linkage-specific antibodies, which specifically recognize Met1, Lys11, Lys27, Lys48 and Lys63 Ub linkages (Matsumoto et al., 2010, 2012; Newton et al., 2012). Ub antibodies have been predominantly utilized to confirm substrate ubiquitination and only to a lesser extent in proteomic studies (Matsumoto et al., 2010, 2012; Newton et al., 2012). The FK2 antibody was the first antibody used for initial MS-based global ubiquitination analysis, which led to identification of 670 Ub substrates and 18 ubiquitination sites in HEK293T cells (Matsumoto et al., 2005). Similar approach resulted in detection of 70 Ub targets from MG132-treated MCF-7 cell line (Vasilescu et al., 2005). Moreover, proteomic analysis of immunoprecipitated Met1-linkages from *Salmonella*-infected HCT116 cells detected 32 putative linear Ub targets (Fiskin et al., 2016). Among advantages of Ub antibodies are detection of ubiquitinated proteins at the endogenous level and a wide applicability, including Western blotting, immunoprecipitation, immunofluorescence and flow cytometry. Since Ub linkage-specific antibodies often exhibit high cross-reactivity, their usage requires proper controls and highly defined experimental conditions (Beaudette et al., 2016).

**FIGURE 7:**
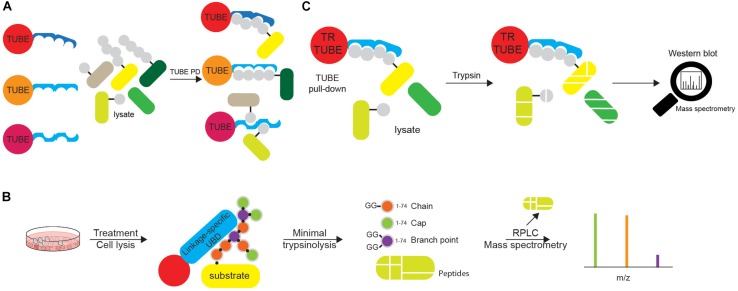
Tandems of ubiquitin entities can be utilized in various ubiquitin tools. **(A)** Tandems of Ub chain-specific or promiscuous UBDs, additionally equipped with affinity tags (such as FLAG-, HA-, GST-, or 6xHIS) and bound to appropriate resins, can be used for affinity purification of ubiquitinated proteins. The use of tandem UBDs increases affinity toward Ub due to avidity, as well as protects ubiquitinated proteins from endogenous DUBs during purification steps. Purified ubiquitinated proteins can be further analyzed by either Western blotting or MS. **(B)** Ub chain enrichment middle-down MS (UbiChEM-MS) approach is based on the enrichment of the specific Ub linkages by linkage-specific UBDs or antibodies, combined with minimal trypsinolysis of Ub that induces a single Ub cleavage after Arg74 and leaves the rest of the Ub molecule intact. By using that approach, Ub molecules within chain, as well as capping and branched Ub conjugates can be detected and quantified by MS. **(C)** Ub-ProT (Ub chain protection from trypsinization) method determines the length of Ub chains bound to target proteins. Trypsin-resistant (TR)-TUBE (i.e., PLIC1 UBA domain lacking Arg residues) is used for the enrichment of ubiquitinated proteins that can be analyzed by Western blotting. Furthermore, since TR-TUBE-protected sample is resistant to trypsin digestion, it can be applied for the determination of the length of Ub chains by quantitative MS.

Yet another technique for identification of Ub-modified proteins and evaluation of their Ub modifications is based on Ub-binding modules. Due to typically low binding affinity of UBDs toward Ub, synthetic multiple repeats of UBDs (tandem Ub-binding entities, TUBEs) were engineered (Figure 7A). Expressed as recombinant epitope-tagged fusions, those tools are characterized by high overall Ub-binding avidity and enable efficient capturing of both high and low abundant Ub targets from cellular lysates (Hjerpe et al., 2009; Mattern et al., 2019). A large number of available Ub traps differs in number and types of UBDs, length of linkers and type of epitope tags. While affinity UBD-based tools are widely used for the confirmation of specific protein ubiquitination, several reports demonstrated their applicability for proteomic analysis of ubiquitinated proteins. Identification of over 290 ubiquitination sites and 223 putative ubiquitinated proteins in HEK293T cells has been demonstrated in ubiquitinome analysis, which used PLIC-1 UBA-based TUBE (Shi et al., 2011). Another proteomic study utilized a tandem of hybrid UBDs (ThUBDs) to analyze total ubiquitinated proteins, which enabled detection of 1092 and 7487 Ub targets in yeast and liver MHCC97-H cell line, respectively (Gao et al., 2016). The recombinant HIS fusion of PLIC2 UBA domain enabled enrichment of polyUb chains from brains of Huntington’s disease model mice, as well as patient samples (Bennett et al., 2007). Moreover, Ub linkage-selective affinity UBD-based probes have also been developed, including Lys29/Lys33 linkage-specific (TRABID NZF-based), Lys63 linkage-specific (TAB2 NZF- and VPS27 UIM-based) and Met1-linkage-specific (UBAN-based) TUBEs (Emmerich and Cohen, 2015). By using tandem VPS27 UIM-based probe, over 100 putative Lys63 Ub-modified proteins were identified in *A. thaliana* (Johnson and Vert, 2016). UBAN-based M1-SUB probe combined with proteomic analysis identified a single linear Ub-modified substrate in THP-1 cells upon NOD2 stimulation (Fiil et al., 2013). Furthermore, the tandem of ZnF UBP domain and hybrid Ub probe comprised of ZnF UBP and UBA domains were designed for isolation of unanchored Ub chains and unconjugated Lys48 linkages, respectively (Scott et al., 2016). To summarize, affinity UBD-based reagents efficiently enrich ubiquitinated endogenous substrates, protect Ub conjugates from DUB-mediated proteolysis and proteasomal degradation and have a wide range of applications, including MS, Western blotting and microscopy (Hjerpe et al., 2009; Shi et al., 2011; van Wijk et al., 2012). However, the use of UBD-based tools for discovery of ubiquitinated substrates requires non-stringent purification conditions that ultimately lead to purification of protein complexes rather than individual Ub-modified proteins. On top of that, some UBDs also bind proteins containing intrinsic UBL domains, which results in a relatively high number of contaminants.

Since neither antibodies nor TUBEs selectively recognizing several atypical Ub linkages were available, David Komander’s group used the affimer technology to screen libraries of small, non-antibody protein scaffolds with randomized surface to develop the linkage-specific Ub affinity reagents for detection of Lys6- and Lys33-linked polyUb chains (Michel et al., 2017). The Lys6 affimer exhibits high selectivity toward Lys6-linked polyUb chains, whereas the Lys33 Ub affinity reagent also recognizes Lys11 linkages. The proteomic analysis of proteins enriched by Lys6 Ub affimer enabled identification of mitofusin-2 and HUWE1 as the Lys6 polyUb-specific substrate and E3 ligase, respectively. Both linkage-specific Ub affinity reagents are suitable for *in vitro* and *in vivo* binding assays, MS, Western blotting and immunofluorescence.

Ub Chain Restriction (UbiCRest) approach is mainly utilized to confirm ubiquitination of putative substrates, which were identified by other methods (Hospenthal et al., 2015). UbiCRest kit provides a set of recombinant DUBs of defined linkage specificities that enable qualitative determination of the type(s) and architecture of Ub linkages modifying protein of interest (Hospenthal et al., 2015). Hitherto, several studies successfully applied UbiCRest, as shown by validation of linear polyUb targets BCL10, CASP8 and TNFR1 (Satpathy et al., 2015; Emmerich et al., 2016; Lafont et al., 2017). However, obtained results highly depend on numerous factors, including reaction conditions, concentration and enzymatic activity of DUBs, incubation period, as well as method used for the enrichment of Ub-modified substrate (Hospenthal et al., 2015).

Although linkage-specific antibodies can be used for studying endogenous polyUb-modified proteins, they cannot clearly distinguish between homotypic and heterotypic Ub chains. Bispecific antibodies detecting heterotypic Ub chains exist so far only for Lys11/LysK48-linked Ub chains (Yau et al., 2017). Complex topology of Ub chains, including branched Ub linkages, prompted the development of novel MS-based approaches for simultaneous detection of multiple modifications on a single Ub moiety. While bottom-up MS (such as Ub remnant profiling) enables characterization of linkages between two Ub molecules, it cannot assess chain length and topology due to trypsin digestion. Opposite to that, middle-down MS utilizes minimal protease digestion of protein samples to detect multiple PTMs on a single Ub molecule (Figure 7B). It is based on the notion that under optimized conditions, native folded polyUb is trypsinized only at the Arg74 residue (Xu and Peng, 2008). In that way, minimal trypsinolysis, by leaving Ub largely intact, enables detection of multiple modifications by MS (Xu and Peng, 2008; Valkevich et al., 2014). Ub chain enrichment middle-down MS (UbiChEM-MS) approach combines the enrichment of specific Ub chains using linkage-specific UBDs with minimal trypsinolysis and middle-down MS for the characterization of branched Ub conjugates (Crowe et al., 2017).

Furthermore, David Komander’s group has recently published Ub-clipping approach that utilizes an engineered viral protease (Lb^pro*^) to incompletely remove Ub from substrates, leaving the C-terminal diGly dipeptide conjugated to the modification site and enabling quantification of multiply diGly-modified branch-point Ub (Swatek et al., 2019). By using that approach they could estimate that around 10–20% of Ub in polymers can be found in branched Ub chains.

The length of substrate-attached Ub chains is usually estimated by monitoring their gel mobility in SDS-PAGE. However, due to the complex nature of ubiquitination (different Ub modifications can be simultaneously attached to a single protein), determining the length of Ub chains is not straightforward. Ub-ProT (Ub chain protection from trypsinization) method (Figure 7C) was recently developed for assessing the length of substrate-attached polyUb chains (Tsuchiya et al., 2018). The method is based on the use of Ub chain protector, i.e. trypsin-resistant (TR)-TUBE, which consists of biotin and 6xHIS tags and six tandem repeats of the PLIC1 UBA domain, in which all the Arg residues are replaced by Ala (to prevent trypsin digestion of the TUBE). When substrate-attached Ub chains are bound by TR-TUBE, they are resistant to trypsin digestion and can be analyzed using a gel-based assay. By combining this method with quantitative MS analysis, Tsuchiya et al. (2018) determined the length and composition of Ub chains in yeast, and of ligand-activated epidermal growth factor receptor (EGFR) in mammalian cells. The observed disadvantage of the Ub-ProT approach is differential protection of various Ub linkages, as some of them (such as Lys6, Lys27, Lys29, and Lys33) are less efficiently protected, similar to decreased protection of branched over homotypic Ub linkages, which will inevitably generate bias in data analysis. The development of novel TR-TUBEs, with a uniform affinity toward all Ub linkages and without preference for homotypic over heterotypic/branched Ub linkages should improve the quality of this approach.

#### Quantification of Ub Modification

In general, quantitative measurements of ubiquitination can be either relative or absolute and involve the use of various labeling approaches, such as metabolic labeling (stable isotope labeling with amino acids in cell culture; SILAC) or isobaric peptide tagging (isobaric tags for relative and absolute quantitation; iTRAQ and tandem mass tags; TMT).

**FIGURE 8:**
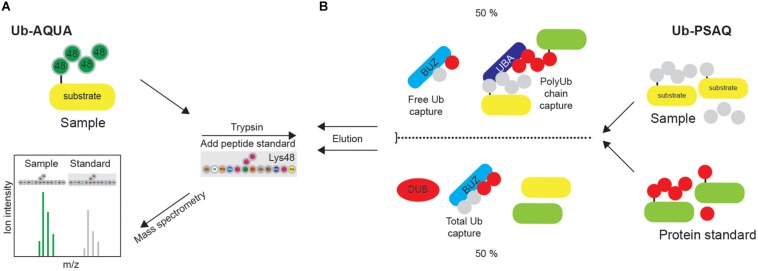
Quantification of ubiquitination *in vivo.* **(A)** Synthetic peptide absolute quantification (Ub-AQUA) MS approach is based on synthetic peptides as quantification standards for both mono- and polyubiquitination. **(B)** Ub-PSAQ approach is based on the use of stable isotope-labeled free Ub and Ub conjugates as protein standards. They are added to lysates and captured with UBD BUZ that is selective for free Ub and UBD PLIC2 UBA (or similar) that recognizes Ub chains. Half of the sample is treated with USP2cc and total free Ub captured by BUZ affinity reagent. Sample is quantified by MS, relative to the peptide standard.

Absolute quantification (AQUA) strategy is often used for absolute quantification of proteins or PTMs. Isotopically labeled synthetic peptide, corresponding to the tryptic peptide of the protein of interest, is used as an internal standard with a known concentration. In the Ub-AQUA approach, all eight ubiquitinated diGly peptides can be labeled with a stable isotope and used as internal standard that can be readily distinguished by MS and used for quantification of corresponding native peptides (Figure 8A) (Kirkpatrick et al., 2005). For examples, Ub-AQUA approach was used to quantify various types of Ub modifications of NEMO, an essential regulator of NFκB signaling (Ikeda et al., 2011).

Since Ub-AQUA approach cannot take into account any experimental loss of protein, Ub-PSAQ approach has additionally been developed. MS-based Ub protein standard absolute quantification (Ub-PSAQ) approach uses stable isotope–labeled free Ub and Ub conjugates as recovery standards, which are added into lysates and captured with affinity reagents either selective for free Ub (ZnF UBP domain that captures unconjugated Ub by interacting with C terminus of Ub) or Ub chains (PLIC2 UBA domain that binds Ub chains) (Figure 8B). Additionally, half of the sample is treated with USP2cc, which enables the conversion of all Ub species to free Ub in order to measure total Ub, and captured by UBD ZnF UBP/BUZ affinity reagent. Sample is subsequently washed, eluted, treated with trypsin and quantified by LC-ESI TOF MS relative to the peptide standard. The presence of DUB inhibitor in the assay prevents interconversion of Ub species during assay (Kaiser et al., 2011).

#### Determination of Cellular Localization of Ubiquitin Modifications

The techniques and approaches for visualizing Ub signals have recently been reviewed in details by van Wijk et al. (2019).

Engineered UBD-based biosensors containing fluorescent tags found important applications for Ub-binding modules in *in vivo* visualization of Ub modifications. The UBAN-based biosensor enabled monitoring of Met1 linkages in TNFα-mediated NFκB signaling and co-localization of linear Ub chains with cytosolic *Salmonella* during xenophagy, whereas Lys63-selective UIM- and NZF-based sensors traced localization and accumulation of Lys63 linkages during DNA damage response (DDR), mitophagy and upon IL-1β and TNF-related weak inducer of apoptosis (TWEAK) stimulation (Sims et al., 2012; van Wijk et al., 2012).

Ubiquitination-induced fluorescence complementation (UiFC) assay is a variation of TUBE-based biosensors. In this technique, visualization of polyUb chains is achieved by expression of two non-fluorescent, complementary fragments of a fluorescent protein fused to UBDs. Upon UBD-mediated binding to polyUb chains in close proximity, the fluorescence of two fragments is restored (Chen et al., 2013; Pinto et al., 2016). As a proof of concept, Lys48-linked Ub chains were visualized with Epsin1 UIM-based UiFC biosensors under various conditions (i.e., mitophagy, proteasome inhibition) (Chen et al., 2013). Importantly, levels of UBD-based biosensors have to be kept low during experiment, as their high expression could restrict activation of cellular signaling pathways (Sims et al., 2012; van Wijk et al., 2012).

Several aforementioned Ub and Ub linkage-specific antibodies (i.e., FK1 and FK2, Lys48- and Lys63-linked polyUb) are suitable for immunocyto- and immunohistochemistry and are widely used (Newton et al., 2008; Danielson and Hope, 2013; Nakazawa et al., 2016). Among others, Met1-linked polyUb-specific antibody was used to determine the effect of linear Ub binding-deficient OPTN mutations in the onset of amyotrophic lateral sclerosis (Nakazawa et al., 2016).

#### Identification of Post-translationally Modified Ubiquitin and Ubiquitin Chains

Discovery of PTMs that modify Ub monomers and polymers has predominantly been achieved by MS. As delineated previously, MS detection of tryptic Ub peptides containing specific modifications enabled identification of Ub PTMs such as deamidation and phosphoribosylation (Cui et al., 2010; Bhogaraju et al., 2019).

The abovementioned Ub-clipping method (Swatek et al., 2019) can also be used to determine the co-existing PTMs on Ub modifications. As a proof of principle, such approach was used to determine Ub architecture on depolarized mitochondria. The analysis revealed that, under mitophagy-inducing conditions, Ub coat on mitochondria is composed mainly of monoUb and oligoUb chains, with phosphoUb capping Ub chains and therefore, preventing further extension of Ub polymers (Swatek et al., 2019).

Currently, there are several available antibodies that detect specific PTMs on Ub. The antibody recognizing phosphorylation on Ub Ser65 is suitable for Western blotting and has been used to study the effect of Ub phosphorylation on the recruitment of Ub-binding mitophagy receptors to depolarized mitochondria (Ordureau et al., 2018). Moreover, the antibody detecting acetylation on Ub Lys48 residue, suitable for ELISA and Western blotting, is available on the market. However, it has not been reported in any publication thus far.

### Methods to Study Ubiquitination Machinery

#### Identification of E3 Ligase Substrates

Identification of E3 ligase:substrate pairs is very challenging, since interactions between E3 ligases and their targets are very dynamic and of low affinity. Moreover, ubiquitination of the substrates often exhibits stimulus- and spatiotemporal dependency. Furthermore, individual substrates can be targeted by several E3 ligases at different residues and at different physiological conditions. Additionally, ubiquitinated substrates are often marked for proteasomal degradation, which leads to their fast removal from the cells.

A plethora of approaches has been established to enable identification of E3 ligase substrates and review by Iconomou and Saunders discusses them in details (Iconomou and Saunders, 2016). These include proximity-dependent biotin labeling (BioID) (Roux et al., 2012; Coyaud et al., 2015), Ub ligase substrate trapping (Mark et al., 2014, 2016; Loveless et al., 2015), Ub-activated interaction traps (UBAIT) (O’Connor et al., 2015, 2018) and NEDDylator approach (Zhuang et al., 2013).

BioID approach allows the identification of proteins in the close vicinity of a protein of interest in living cells. It is based on the fusion of the E3 ligase with mutated form of biotin ligase BirA, which biotinylates all the proteins in the close vicinity (around 10 nm), if biotin is available. Such “neighborhood tagging” allows AP and subsequent MS-based detection of all the labeled proteins, majority of which potentially being the E3 ligase substrates. This approach enabled identification of 50 putative substrates of SCF^βTrCP1/2^ (Coyaud et al., 2015).

**FIGURE 9:**
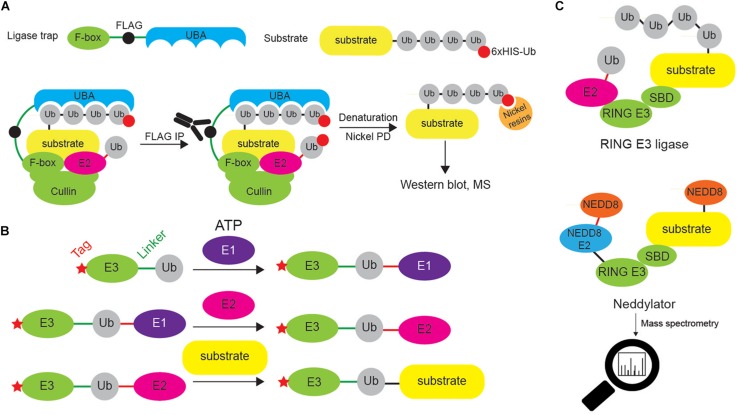
Different approaches to identify E3 ligase substrates. **(A)** Ligase trapping approach “stabilizes” E3 ligase:substrate non-covalent interactions by UBDs (such as UBA domain) fused to E3 ligase substrate-interacting domains (such as F-box of the multi-protein E3 ligase complex SCF). When such ligase traps and 6xHIS-tagged Ub are overexpressed in cells, the UBA interacts with the nascent Ub chain on endogenous SCF substrates, thereby delaying their release. Cells are then lysed and subjected to an anti-FLAG coimmunoprecipitation under native conditions, to isolate ligase trap complexes (FLAG tag is inserted between F-box and UBA). FLAG eluates are then used in denaturing Ni-NTA agarose pull-down to exclusively enrich ubiquitinated substrates (and to remove any non-covalently interacting proteins). **(B)** UBAITs, similar to ligase traps, enable identification of E3 ligase substrates (for both HECT and RING E3 ligases), as well as their adaptors and regulators. Unlike ligase traps, UBAITs are fusions of N-terminal affinity-tagged E3 and C-terminal Ub molecule. With the help of cellular E1 and E2, UBAIT E3 component transfers UBAIT Ub component (by forming amide bond) to proteins that interact with the E3, such as E3 ligase substrates. Formed complex is easily affinity purified and analyzed by mass spectrometry. For HECT E3s, both E3 and E2 thioester-linked interacting proteins can be captured by UBAITs. **(C)** NEDDylator is a catalytic tagging tool, in which Ubc12, an E2 enzyme for NEDD8, is fused to an E3 ligase substrate-binding domain, allowing for the transfer of NEDD8 to the E3 substrate, and MS-based identification of E3 ligase-target pairs.

Ligase trapping is an AP approach in which E3 ligases fused to UBDs are used for isolation of ubiquitinated substrates (Figure 9A). The presence of UBD increases the binding affinity of the E3 ligase of interest toward its targets, thus increasing sensitivity of the method. This technique enabled successful identification of novel substrates of FBXL E3 ligases, including Prb1 (Mark et al., 2014). The selection of proper UBD (to ensure effective enrichment of substrates), as well as fusion point (that might potentially disrupt the substrate recruitment) is essential for the proper functionality of the ligase trap.

UBAIT is a method belonging to ligase trapping class and allows for identification of substrates for HECT and RING E3 ligases (Figure 9B). UBAIT tool consists of E3 ligase fused to Ub moiety and target-interacting domain. The presence of Ub enables E1- and E2-mediated activation of UBAIT and subsequent covalent capture of E3 ligase substrates. This technique was applied to identify proteins interacting with several Ub ligases, such as ITCH and RNF126 (O’Connor et al., 2015). The drawback of this approach is that it cannot distinguish between E3 ligase substrates and E3 ligase-interacting proteins.

NEDDylator approach relies on the fusion between NEDD8 E2 enzyme and substrate-binding region of desired E3 ligase (Figure 9C). Such configuration allows artificial NEDDylation of endogenous E3 ligase substrates, their enrichment (by denaturing immunoprecipitation of exogenous NEDD8 tag, such as 6xHIS) and subsequent MS identification. As NEDDylation does not occur at a high level in a cell, it is not difficult to distinguish between endogenous and NEDDylator-induced modifications and as such, to identify E3 ligase substrates.

#### Identification of DUB Substrates

Identification of DUBs and their targets is difficult due to several reasons: the enzymatic activity of DUBs results in a rapid removal of Ub modifications from the DUB substrates, the interaction between DUBs and their substrates is often inducible and spatiotemporally restricted, DUBs typically bind their substrates with relatively low affinity and numerous DUBs require accessory proteins for specific interactions with their targets. Therefore, a limited number of studies have aimed to identify the substrates for specific DUBs thus far.

A common technique is substrate AP with either recombinant or ectopically expressed epitope-tagged DUB as bait (Bonacci et al., 2018). However, this approach preferentially identifies DUB interactors over DUB substrates. If known, point mutation of the active site of investigated DUB, which decreases/abolishes the proteolytic activity of the DUB, greatly facilitates identification of DUB targets as it enables entrapment of ubiquitinated substrates. This approach led to the identification of APC/C substrates (i.e., Cyclin B and Aurora A) as targets of Cezanne/OTUD7B, a Lys11 linkage-specific DUB (Bonacci et al., 2018). Another study utilized similar approach to identify NFX1-123 as a substrate of USP9X (Chen et al., 2019).

Yet another method for discovery of DUB targets is based on *in vitro* deubiquitination of cell lysate with recombinant DUB of interest. Together with reference lysate, samples are then digested with trypsin, peptides are isotopically labeled and subjected to Ub remnant profiling. This quantitative proteomic approach enabled identification of two substrates of *Salmonella Typhimurium* effector SseL (Nakayasu et al., 2015).

#### Measuring the Enzymatic Activity of Ubiquitination Machinery

E3 ligases and DUBs have evolved in the last several years as promising therapeutic targets in oncology and neurodegeneration, as they are often perturbed in various diseases and cancer types (Cromm and Crews, 2017; Harrigan et al., 2018; Yang et al., 2018). Many research groups and pharmaceutical industry are therefore developing specific inhibitors and activators of these enzymes, as well as improving and developing quantitative methods for measuring their enzymatic activity both *in vitro* and *in vivo*. A comprehensive review about activity-based probes (APBs) for ubiquitination machinery has recently been published by Huib Ovaa’s group (Witting et al., 2017).

Initially developed ABPs contain Ub moiety with the C-terminal Gly76 residue chemically modified with an electrophilic warhead, such as aldehyde, vinyl sulfone (Ub-VS), vinyl methylester (Ub-VME) and propargylamide (Ub-Prg) to covalently bind proteins containing active Cys residue (Borodovsky et al., 2001, 2002; Ekkebus et al., 2013). They have been successfully used to identify novel DUBs and monitor DUB activity (Borodovsky et al., 2002; de Jong et al., 2012). The real advancement in the field came after the successful diUb chemical synthesis, which opened new possibilities in developing DUB probes (El Oualid et al., 2010; de Jong et al., 2012; Mulder et al., 2014; Flierman et al., 2016). Many approaches have also been developed for assessing DUB specificity, ranging from diUb probes mimicking all eight different Ub linkages combined with MS (McGouran et al., 2013), diUb probes resembling native diUb that contain a Michael addition acceptor for trapping the DUB active-site Cys (Li et al., 2014), seven synthetic isopeptide-linked diUb FRET probes with rhodamine-TAMRA for the absolute quantification of chain cleavage specificity (Geurink et al., 2016) or monitoring total cellular DUB activity by advanced chemoproteomics (Pinto-Fernandez et al., 2019).

Many assays for assessment of E3 ligase activity are based on monitoring E3 ligase autoubiquitination, either by Western blotting or by measuring fluorescence. By combining time-resolved fluorescence resonance energy transfer (TR-FRET) based on lanthanide chemistry and TUBEs, Marblestone et al. (2012) have developed an E3 ligase activity assay in which they monitored the proximity of the autoubiquitinated E3 ligase and biotinylated TUBEs in an E3-dependent polyUb chain formation assay based on endogenous Ub. The method is limited to studying E3 ligase activity of the specific, individual E3 ligase *in vitro* and might not work in cellular lysates. For E3 ligases that require PTMs or additional protein components for their enzymatic activity such approach can be technically very challenging.

The development of E1-E2-E3 activity probes was not as fast as the development of DUB probes. Some ABPs, such as Ub-Prg and Ub-VME, can also label active Cys in HECT E3 ligases (Ekkebus et al., 2013).

Recently published cascading E1-E2-E3 ABP (Mulder et al., 2016) is based on the use of the Ub variant in which Gly76 is replaced by dehydroalanine (Dha) that can be processed by the cellular ubiquitination machinery (Hodgins et al., 1992; Pickart et al., 1994; Mulder et al., 2016). Once added to lysate or electroporated into cells, UbDha is activated by E1 enzyme (through the formation of an adenylate intermediate) and the activated reactive methylene group of the Dha moiety can then either covalently trap the enzyme in an E1-UbDha thioether adduct or follow the native resulting in an E1∼UbDha thioester. Such thioester can then be transferred to an E2 enzyme and either form covalent thioether adduct with the probe or undergo native trans-thioesterification. Following the ubiquitination pathway UbDha can subsequently be transferred to an active site of either HECT or RBR E3 ligases.

In this manner, the probe can travel through the entire E1-E2-E3 cascade, where it “traps” catalytically active Ub-modifying enzymes along the way. Unlike endogenous Ub, the probe can irreversibly react with the active site Cys residue of target enzymes in living cells. It can be also combined with MS to identify or quantify E1-E2-E3 cellular activities. However, in its current form the probe is not selective for specific E2 or E3 enzymes and cannot capture RING E3 ligases in a mechanism-dependent manner (Mulder et al., 2016), which requires novel probe designs.

Pao et al. (2018) have recently developed a novel ABP consisting of Ub-charged E2 conjugate with C-terminal activated vinylsulfide (E2-Ub-AVS) as the warhead for the detection and identification of novel E3 ligases. By using their probe, they identified MYCBP2/PHR1 as so far unique E3 ligase with esterification activity and intrinsic selectivity for Thr over Ser (Pao et al., 2018). Like E1-E2-E3 probe, this approach also lacks the ability to study specific E3 ligases and to monitor RING E3 ligase family.

Additionally, Dha-based E2-Ub ABP was also developed for monitoring HECT E3 activity *in vitro* and *in vivo* (Xu et al., 2019).

The UPS-confocal fluorescence nanoscanning (UPS-CONA) assay is based on the immobilization of the specific substrate of interest on micro-beads. Fluorescently labeled Ub is enzymatically conjugated to the substrate and can be quantitatively detected on the bead periphery by confocal microscopy. UPS-CONA approach can be used for studying specific enzymes of the ubiquitination machinery, as well as for measuring the selectivity of putative ubiquitination inhibitors (Koszela et al., 2018).

### Methods to Study Non-covalent Ubiquitin Recognition

#### Identification of Novel Ubiquitin Readers and Determination of Their Specificity Toward Ubiquitin

The number of currently known Ub readers is relatively low in comparison to the number of proteins playing active roles in ubiquitination and deubiquitination or those modified by various Ub moieties. It is reasonable to speculate that there are still multiple UBDs that remain unknown at the moment, especially those specific for Ub linkages, those that are Ub chain length-dependent or can specifically recognize heterotypic/branched Ub linkages or even specific Ub PTMs.

**FIGURE 10:**
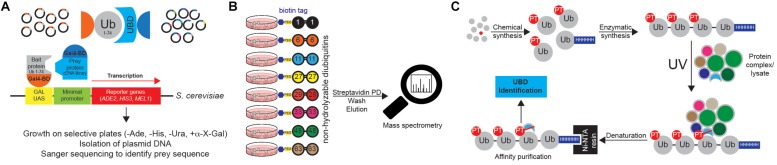
Approaches to identify ubiquitin receptors. **(A)** A very common type of Y2H approach is based on the use of Ub sequence (lacking C-terminal Gly-Gly motif) as N-terminal fusion with the GAL4 binding domain (BD). The cDNA library containing putative UBDs consists of cDNAs cloned under the control of the lacZ promoter downstream of the DNA sequence encoding the activating domain (AD) of the yeast GAL4 transcription factor. Protein interaction induces the close proximity of GAL4AD and GAL4BD, forming the active transcription factor, which binds to the GAL1 upstream activating sequence (UAS) and activates the transcription of several GAL4-responsive genes, which are used as reporters. For example, yeast strain *S. cerevisiae* YTHGold (*Clontech*) enables very stringent quadruple selection, since it contains 4 reporters. In that way background growth and detection of false positive interacting proteins is significantly decreased, simplifying further evaluation steps. **(B)** UbIA-MS method relies on the use of 8 chemically synthesized non-hydrolyzable biotinylated diUbs that can be used for *in vitro* affinity purification of Ub interactors. These diUb linkages mimic native diUb, and have advantage of not being cleaved by cellular DUBs, which prevents the loss of captured material and decrease in Ub chain specificity. Upon purification, samples are digested on beads with trypsin, followed by liquid chromatography (LC)-MS/MS analysis. **(C)** Ub-PT is a synthetic Ub variant that contains a photo-activatable crosslinking Leu mimic photoleucine (pLeu) at positions 8 or 73 in Ub molecule. Importantly, these modifications do not affect Ub functionality, including its ability to bind UBDs. Enzymatic polymerization of Ub-PT into Ub chains of defined lengths and linkage types allows the use of Ub-PT as UV-activatable crosslinking reagent (phototrap) for irreversibly capturing Ub receptors. Furthermore, the existence of 6xHIS tag in Ub-PT-containing reagents allows stringent isolation of Ub interactors, without co-purification of their binding proteins.

The use of single Ub moiety lacking diGly motif at the C terminus (to prevent potential conjugation to yeast proteins) as bait in yeast two-hybrid (Y2H) screen led to the identification of several novel UBD-containing proteins, including Pru domain of RPN13 (Figure 10A) (Bienko et al., 2005; Husnjak et al., 2008; Wagner et al., 2008). This approach is limited to UBDs that bind single Ub moieties and cannot be used for Ub linkage-specific UBDs.

Ub chains cannot be efficiently used for the AP of Ub chain-specific UBDs, as cellular DUBs readily cleave them. However, by combining chemically synthesized non-hydrolyzable diUb molecules with AP/MS, Zhang et al. (2017) could successfully enrich and identify many known and novel Ub interactors with simultaneous evaluation of their Ub linkage specificity (Figure 10B). The novel method, termed Ub interactor affinity enrichment-MS (UbIA-MS) identified TAB2 and TAB3 as novel Lys6 interactors and characterized UCHL3 as Lys27-specific Ub receptor (Zhang et al., 2017).

Another promising approach for identifying novel UBDs is the use of synthetic Ub variant that contains a photoactivatable crosslinking side chain. Photoleucine (pLeu) incorporated into fully synthetic Ub monomer does not prevent UBD binding to its Ile44 patch and can be readily incorporated into polyUb chain, without affecting the specificity of the binding (Figure 10C). Once photoactivated by UV, it is able to crosslink nearby proteins, thus stabilizing often very weak UBD:Ub interactions. By using Ub-PT as a tool, Chojnacki et al. (2017) identified proteasomal subunit Rpn1 as a novel Ub receptor. Moreover, Ub-PT can have pLeu incorporated at multiple positions within Ub molecule, making it very useful for capturing UBDs that interact in different ways with Ub.

## Concluding Remarks

Ub field has been extensively studied in the last several decades, and its recognition as the promising drug target has initiated a large number of studies aiming to improve our understanding of the complex nature of ubiquitination regulation. Novel DUB inhibitors have been developed, as well as multiple tools to identify novel components (enzymes, scaffolds, and receptors), to study enzymatic activity, cellular distribution, modes of regulation and potential chemical inhibition of Ub system.

The use of proteolysis targeting chimeras (PROTACs) for induction of specific protein degradation has emerged as promising approach for targeting proteins that are otherwise hard or impossible to target by small molecule approaches (Watt et al., 2019), making further research of the Ub system a priority. Still, there are many things that we do not know or that we have just started to elucidate. Newly developed tools and approaches will clearly shed a new light on our understanding of Ub systems and ways how we can explore it for treating diseases.

## Outstanding Questions

There are still no straightforward approaches for determining E3 ligases responsible for specific ubiquitination events. Furthermore, ABPs specific for single E3 ligases (and many DUBs) are still missing, as well as small molecules that can either specifically inhibit (or potentially activate) these enzymes.

Even though numerous high-throughput MS studies have identified tens of thousands of ubiquitination sites at the proteome level (Kim et al., 2011; Wagner et al., 2011), for only a small proportion of these modifications a specific E3 ligase is known. Methods such as Ub remnant profiling do not allow identification of E3 ligases that modify specific Lys residues on identified proteins.

Due to transient interaction between E3 ligases and their substrates, standard approaches (such as immunoprecipitation) are not appropriate for identification of specific E3 ligases:substrate pairs, even more so since many (but not all) E3 ligases target their substrates for proteasomal degradation, thus reducing their levels. In line with that, protein abundance upon E3 ligase removal/inhibition or activation/overexpression cannot be a good readout for identifying specific E3 ligase substrates.

As many E3 ligases are multicomponent complexes, whose activation often depends on various PTMs, many of these enzymes are difficult to be used in various high-throughput screening efforts for their regulators. As ubiquitination is a very complex PTM, with numerous heterotypic/branched and combinatorial ubiquitination events, it is clear that all the available methodology is still unable to fully comprehend the extent and importance of these modifications.

## Author Contributions

Both authors listed have made a substantial, direct and intellectual contribution to the work, and approved it for publication.

## Conflict of Interest

The authors declare that the research was conducted in the absence of any commercial or financial relationships that could be construed as a potential conflict of interest.
